# Heartbeat evoked potentials and autonomic arousal during dissociative seizures: insights from electrophysiology and neuroimaging

**DOI:** 10.1136/bmjno-2024-000665

**Published:** 2024-06-05

**Authors:** Vera Flasbeck, Johannes Jungilligens, Isabell Lemke, Jule Beckers, Hilal Öztürk, Jörg Wellmer, Corinna Seliger, Georg Juckel, Stoyan Popkirov

**Affiliations:** 1 Division of Clinical and Experimental Neurophysiology, Department of Psychiatry, Psychotherapy and Preventive Medicine, Ruhr University, LWL University Hospital, Bochum, Germany; 2 Department of Neurology, Ruhr University, University Hospital Knappschaftskrankenhaus, Bochum, Germany; 3 Faculty of Psychology, Ruhr University, Bochum, Germany; 4 Ruhr Epileptology, Department of Neurology, Ruhr University, University Hospital Knappschaftskrankenhaus, Bochum, Germany; 5 Department of Neurology, University Hospital Essen, Essen, Germany

**Keywords:** FUNCTIONAL NEUROLOGICAL DISORDER, EEG, EVOKED POTENTIALS, SOMATOSENSORY, MRI, STRESS

## Abstract

**Introduction:**

Dissociative seizures often occur in the context of dysregulated affective arousal and entail dissociative symptoms such as a disintegration of bodily awareness. However, the interplay between affective arousal and changes in interoceptive processing at the onset of dissociative seizures is not well understood.

**Methods:**

Using retrospective routine data obtained from video-electroencephalography telemetry in a university hospital epilepsy monitoring unit, we investigate ictal changes in cardiac indices of autonomic arousal and heartbeat evoked potentials (HEPs) in 24 patients with dissociative seizures.

**Results:**

Results show autonomic arousal during seizures with increased heart rate and a shift towards sympathetic activity. Compared with baseline, ictal HEP amplitudes over central and right prefrontal electrodes (F8, Fz) were significantly less pronounced during seizures, suggesting diminished cortical representation of interoceptive information. Significant correlations between heart rate variability measures and HEPs were observed at baseline, with more sympathetic and less parasympathetic activity related to less pronounced HEPs. Interestingly, these relationships weakened during seizures, suggesting a disintegration of autonomic arousal and interoceptive processing during dissociative seizures. In a subgroup of 16 patients, MRI-based cortical thickness analysis found a correlation with HEP amplitudes in the left somatosensory association cortex.

**Conclusions:**

These findings possibly represent an electrophysiological hint of how autonomic arousal could negatively impact bodily awareness in dissociative seizures, and how these processes might be related to underlying brain structure.

**Key messages**

- Alterations in the interoceptive processing of arousal potentially play a key role in dissociative seizures but are not well understood.
- This study finds that while sympathetic arousal is heightened during seizures, electroencephalography markers of interoceptive processing are reduced.
- These findings indicate how autonomic arousal could negatively impact bodily awareness in dissociative seizures.

## Introduction

Dissociative seizures, also known as functional or psychogenic nonepileptic seizures, are paroxysms of impaired awareness and behavioural control that include dissociative symptoms and stereotypical movement patterns and often occur in the context of dysregulated affective arousal.^1 2^ Although there is considerable heterogeneity in the clinical symptoms of dissociative seizures, studies have found a range of group-level abnormalities of arousal physiology associated with dissociative seizures, for example, increases in respiratory rate, sweating and preictal stress hormone levels.^3–6^ One prominent index of autonomic arousal at seizure onset that has often been investigated is the heart rate (HR) and its variability (HRV): ECG studies have shown cardiosympathetic activation just before or during dissociative seizures^7–10^ with small to moderate effect sizes when comparing pre-ictal to ictal HRs,^11^ and some studies did not find alterations in peri-ictal HR at all.^12 13^

Clinically, about 40% of patients report noticing increases in HR in association with their dissociative seizures.^4 14^ Studies using self-report measures find signs of autonomic arousal of up to 89%.^15^ Patients who do not report ictal heart racing might simply not have such signs of arousal or might be unable to recollect the seizure experience (dissociative amnesia), but they might also have limited awareness of or insight into their bodily sensations: Interoceptive accuracy, the ability to monitor and evaluate the internal state of the body correctly and precisely, has been found to be reduced in dissociative seizures in some (but not all) studies.^16 17^ A recent study showed that experimental induction of dissociation reduced interoceptive accuracy in patients with dissociative seizures and other forms of functional neurological disorder (FND).^18^ This suggests that interoception might be closely related to dissociation, which is the disruption of higher-order cognitive functions such as integrated perception, body representation and behavioural control. Dissociation is highly prevalent in FND and one of the defining features of dissociative seizures. Besides guiding biophysiological aspects such as the regulation of the body, interoceptive information about the state of the body is also fundamental to the experience of the self, as it is related to aspects of body awareness and body ownership.^19^ This means that the signals arising within the body shape how we perceive ourselves, become aware of our bodies and recognise them as our own.

While interoception mostly operates outside of awareness, some aspects can be assessed using more objective measures. For example, heartbeat evoked potentials (HEPs) are understood to reflect cortical processing of cardioceptive signals. HEPs are visible in electroencephalography (EEG) as evoked potentials when averaging the EEG signal time-locked to the R-spike of the heartbeat. HEPs can be used to investigate cortical representation of interoceptive information, as they are not dependent on the conscious perception of heartbeats^20^ but are altered in amplitude when alterations in interoceptive processing occur (eg, momentarily through shifting attention to/from the heartbeat or chronically in neuropsychiatric illness^21 22^).

In summary, HR characteristics and associated HEPs could offer an interesting electrophysiological window into the neural interplay between affective arousal and changes of bodily awareness at the onset of dissociative seizures. In a recent retrospective study examining the EEG of 25 patients with dissociative seizures, average HEP amplitude was significantly reduced over right frontal and central electrodes when comparing preictal to interictal states.^23^ The authors of this study interpreted this first evidence of HEP alterations as a neurophysiological marker of aberrant interoception during the onset of dissociative seizures. It is unclear whether these HEP alterations are secondary to downstream changes in cortical processing in a state of dissociation, or whether they are more closely related to early preictal arousal. Surprisingly, the relationship between HEP and autonomic arousal has been rarely examined. In two studies in patients with derealisation/depersonalisation disorder and borderline personality disorder, the amplitude of HEPs was larger with lower salivatory cortisol and with predominance of parasympathetic influence on HRV.^20 24^ In healthy volunteers, experimentally induced arousal has been shown to induce subtle but statistically significant changes in HEPs.^25^

In the search for neuroanatomical correlates of alterations in interoception, correlations between HEPs and brain structure—namely grey matter volumes of regions associated with interoceptive processing—have been found in patients with borderline personality disorder.^26^ Specifically, in a region-of-interest analysis, positive correlations were found between the HEP amplitude and the left anterior insula and bilateral dorsal anterior cingulate cortex. This was interpreted as a neural correlate of low-level registration of afferent interoceptive signals.

A systematic analysis of the interplay between autonomic arousal, interoception and dissociation could potentially disentangle the neurophysiology underlying dissociative seizures. In preparation of such an investigation, we conducted a pilot study of archived electrophysiological recordings before and during dissociative seizures, in which HRV and HEP were analysed to understand the association between ictal interoceptive processing and autonomic arousal. Based on previous results from studies reporting heightened autonomic arousal^3 4 7–10^ and alterations in HEPs,^23^ we hypothesised an increase in autonomic arousal (HR and HRV) and a reduction in the cortical representation of interoceptive information (HEPs) at the start of dissociative seizures compared with baseline. Besides these hypothesis-guided analyses, we furthermore aimed to explore the relationship between these alterations in arousal and interoceptive processing, and the relationship between HEPs and cortical thickness.

## Methods

### Sample selection and characteristics

**Figure 1:**
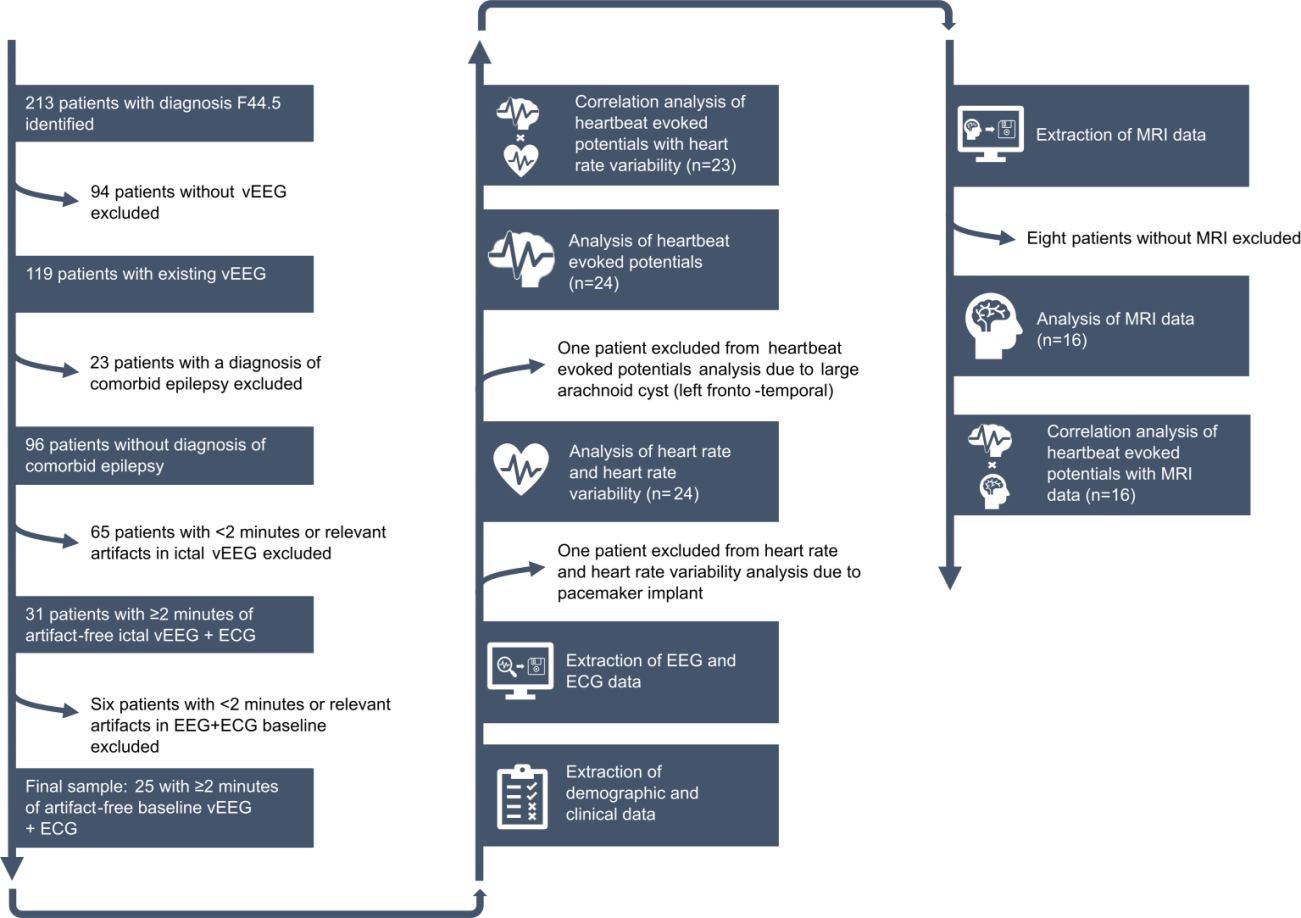
Flow chart depicting the search and selection process as well as the data analysis plan. vEEG, video-electroencephalography.

Patients were identified through a search in the electronic database (June 2015–April 2023) of the Ruhr Epileptology, a level 4 epilepsy centre that is part of the Department of Neurology at the University Hospital Knappschaftskrankenhaus Bochum, Germany. All patients with the final diagnosis of dissociative seizures (F44.5) were selected and cross-referenced with additionally available electronic lists from previous studies.^17 27–29^ This resulted in an initial sample of 213 patients (208 from the hospital’s electronic database, 5 additionally identified from other studies) fulfilling the search criteria. These were then checked for the following criteria: availability of video-EEG (vEEG), no diagnosis of comorbid epilepsy, availability of ≥2 min of artifact-free ictal vEEG (EEG+ECG) and availability of ≥2 min of artifact-free baseline vEEG (EEG+ECG). Baseline was defined as immediately before the onset of the seizure, or (if not available) as interictally from the same day. Figure 1 displays the search and selection process.

The final sample for the HEP analysis consisted of 25 patients. Demographic and clinical characteristics (age, gender, illness duration, medication and seizure semiology of indexed event) were extracted from electronic records, see online supplemental table 1 for details. Mean age was 34.5 years (range 17-66 years); 17 patients were female and 7 male (including 1 identifying as transmale). Mean illness duration was 6.2 years (SD 7.2 years). Seizure frequency was daily in 6, weekly in 4, monthly in 4 and yearly in 1 (binning into categories was performed for this study); in 10 patients, seizure frequency could not be quantified from case notes. Due to the nature of the study, all patients fulfilled the highest level of diagnostic certainty (‘documented’ following established criteria).

10.1136/bmjno-2024-000665.supp1 Supplementary data

In those patients with available MRI data (n=16), mean age was 40.2 years (range 18-66), 10 patients were female and 6 male (including one transmale). Mean illness duration was 6.6 years (SD 8.2 years), seizure frequency was daily in five, weekly in three and monthly in two; in six patients, seizure frequency could not be quantified from case notes.

### Segmentation of baseline and Ictal electrophysiology

vEEG data (electrode locations according to the standard 10–20 system; also including ECG channels) were segmented into baseline (2 min immediately before seizure onset or, if not available, interictally from the same day) and ictal recordings (a minimum of 2 and a maximum of 5 min starting at seizure onset). In contrast to epileptic seizures, where the onset of a seizure can be identified based on characteristic EEG changes, this is not possible with dissociative seizures. Patients can report subjective sensations preceding the ‘actual’ seizure, but there is no valid rationale for delineating ‘prodromal’ from ‘actual’ phenomena. For our analysis, we defined seizure onset either at the time of the patient’s report of seizure onset (via bedside button or verbally) or at the time of the first objectifiable manifestation in the video clearly attributable to a seizure.

### Analysis of cardiovascular data

ECG data were analysed using the software Kubios HRV Premium 3 (V.3.1, Kubios Oy, Kuopio, Finland). We extracted the results of the mean HR, the SD of normal-to-normal intervals (SDNN; a measure of the variability between normal heartbeats indicating overall autonomic nervous system activity) and the root mean square of the successive differences (RMSSD; a measure of short-term variability in HR reflecting parasympathetic activity). In addition, we exported the SNS index (index for the activation of the sympathetic autonomic nervous system) and the PNS index (respective index for parasympathetic autonomic nervous system). For detailed information on index calculation, see Flasbeck *et al*.^21^ PNS or SNS index values of 0 indicate that the parameters are equal to the normal population average. Therefore, indices below or above 0 show how many SD the participant is deviating from the normal population. Data of one participant were excluded from further HRV analysis because of a pacemaker implant.

### Analysis of HEPs

For analysis of HEP, BrainVision Analyzer (V.2.2.2; Brain Products, Germany) was used. Following established practices,^30^ all EEG recordings were downsampled to 256 Hz and re-referenced offline to the average earlobes. Data were filtered with a 50-Hz-notch and band pass filters (0.1-35, 24 dB/octave roll-off Hz). The recordings were visually inspected for muscle and technical artefacts. Eye movements were removed manually by independent-component analysis. EEG data were segmented according to the R-waves detected in the ECG signal (-125-25 ms before R-wave until 800 ms following R-wave). Baseline correction for -125-25 ms before the R-wave was conducted. Artefacts exceeding±100 µV were excluded and segments were averaged for each participant. For baseline conditions, 176.5 (SD=47.7) segments were averaged and for seizure conditions, 190.3 (SD=113.5) segments were averaged.

As in previous studies, we extracted the mean amplitudes in the time frame of 455–595 ms after the R-wave and 250-455 ms after the R-wave.^30^ In the meta-analysis of Coll *et al*, the early time frame (including time frames covering 200-300 ms post R-wave) has been reported to be related to HEPs during arousal, whereas differences between control and clinical groups were found for the later time frame (including 400-500 ms time frames).^30^ Even if no control group has been included in this study, we analysed the later time frame due to findings showing an impact of depersonalisation/derealisation and cortisol on HEPs in the later time frame.^31^ Mean HEP amplitudes of the frontal (F7, F8, Fz) electrodes were selected for further analyses. Results concerning additional electrodes F3, F4, Fp1, Fp2, Fpz, C3, C4 and Cz are shown in online supplemental material. To check whether confounding cardiac effects contribute to HEP differences between conditions, we additionally analysed the ECG mean amplitude in both time frames.

### Analysis of cortical thickness data

To prepare cortical thickness analyses, the FreeSurfer 7.4.1 recon-all-clinical pipeline was used. This new tool enables performing the FreeSurfer cortical reconstruction on clinical-grade MRI scans irrespective of imaging quality.^32^ Outputs were visually inspected for quality and accuracy. Prior to statistical analyses, a 10 mm full width at half-maximum Gaussian Kernel was applied to cortical thickness maps. See online supplemental material for preprocessing details.

### Statistical analysis

Statistical analysis of HRV and HEP data was performed by using SPSS V.29 (IBM). For analysis of HRV, dependent t-tests were used. Repeated-measures analysis of variance (ANOVA) with the factors condition (baseline vs seizure), time frame (early time frame 250-455 ms vs later time frame 455-595 ms) and electrode (F7, F8 and Fz) were calculated to analyse HEP data. ANOVAs for additional electrodes were calculated tor electrode sets F3, F4 and Fz, Fp1, Fp2 and Fpz and for C3, C4 and Cz (see online supplemental material). For post hoc comparisons, paired t-tests were used. For control analysis of ECG signals, repeated-measures ANOVA with the factors condition (baseline vs seizure) and time frame (early time frame 250-455 ms vs later time frame 455-595 ms) were used. Partial η² and Cohen’s *d* are reported as measures of effect sizes. Finally, for correlational analyses between HRV and HEP data, partial correlation coefficients were calculated for the early time frame, controlling for ECG signals. Bonferroni correction for multiple comparisons was applied. To test whether correlation coefficients differ between conditions, Fisher r-to-z transformations were calculated and compared between conditions (one sided). Moreover, the differences between baseline and seizure (Δ=baseline-seizure) were calculated for HRV and HEP data and correlations between Δ of HEPs and Δ of HRV markers were computed.

Whole-brain/whole-hemisphere vertex-vise cortical thickness correlation analyses with HEP amplitudes were performed using single-class GLM analyses, corrected for multiple comparison using a Monte Carlo simulation with a cluster-wise threshold of p<0.05. All analyses were two tailed, controlled for age, sex and total intracranial volume. We furthermore controlled for scanner type to correct for potential effects of different scanners and scanning protocols. Analyses were performed hemisphere-wise, for example, F7-HEPs with left-sided cortical thickness, F8-HEPs right-sided values; Fz-HEPs were correlated across both hemispheres.

## Results

### HR variability

HRV analysis showed ictal changes across parameters in line with a sympathetic arousal reaction (table 1). Statistically significant increases in HR and SDNN were seen from baseline to seizure along with a decrease of the PNS index. The increase in SNS index was visible at trend level (d=-0.38, p=0.079).

**Table 1:** Comparison of heart rate variability measures between baseline and seizure recordings

Measure	Baseline	Seizure	t-test
HR (bpm)	76.91±11.26	87.72±15.28	t(23)=-4.83, p<0.001, d=-0.99
PNS index	-0.88±1.02	-1.31±1.02	t(23)=2.43, p=0.023, d=0.50
SNS index	1.66±1.35	2.25±1.62	t(23)=-1.84, p=0.079, d=-0.38
SDNN (ms)	44.69±17.26	63.87±25.55	t(23)=-4.56, p=0.001, d=-0.93
RMSSD (ms)	32.17±17.97	36.04±19.78	t(23)=-1.05, p=0.304, d=-0.21

HR, heart rate; PNS, parasympathetic autonomic nervous system; RMSSD, root mean square of the successive differences; SDNN, SD of normal-to-normal intervals; SNS, sympathetic autonomic nervous system.

### Heartbeat-evoked potentials

**Figure 2:**
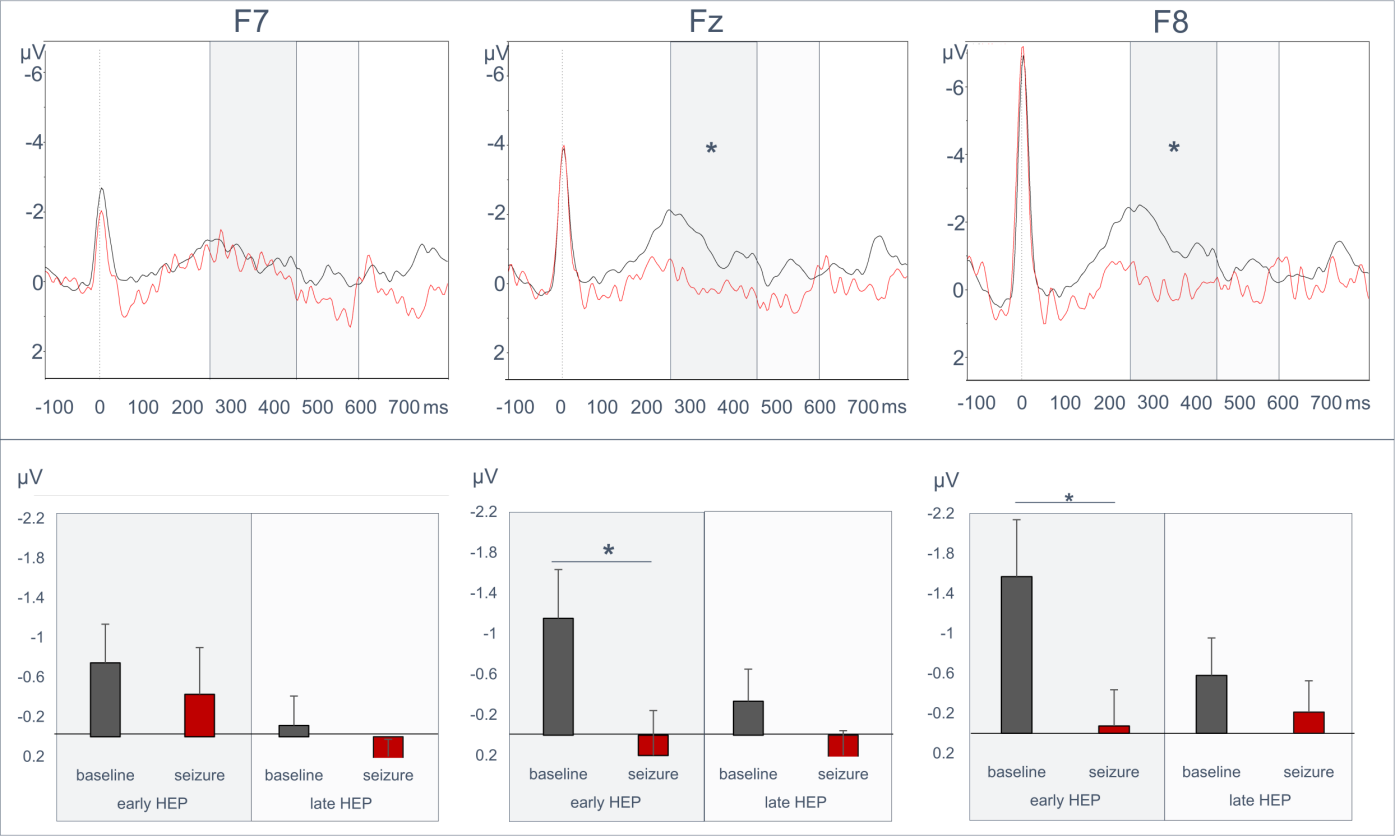
Comparisons of heartbeat-evoked potential (HEP) waveforms (upper row) during baseline (black) and seizure (red) for F7 (left), Fz (middle) and F8 (right) electrodes. Mean HEP amplitudes and SEM (SE of the mean) are shown in bar diagrams in the lower row. *p<0.05.

The ANOVA revealed a main effect of time frame (F(1,23)=11.49, p=0.002, partial η²=0.338) and an interaction of time frame with condition and electrode (F(1.33, 30.68)=7.58, p=0.006, partial η²=0.248). As expected, the main effect of time frame shows a difference in HEP amplitude between time frames (250-455 ms after R-wave: M=-0.63, SD=1.40; 455-595 ms after R-wave: M=-0.03, SD=1.07; t(23)=-3.42, p=0.002, d=-0.699). Interestingly, the interaction showed that smaller amplitudes (less negative values) were found during seizures compared with baseline recordings (figure 2) for electrodes F8 and Fz (F8 baseline M=-1.57 μV, SD=2.78, seizure: M=-0.07 μV, SD=1.77; t(23)=-2.30, p=0.031, d=-0.469; Fz baseline: M=-1.15 μV, SD=2.36, seizure: M=0.20 μV, SD=2.16; t(23)=-2.285, p=0.032, d=-0.466; F7 baseline: M=-0.74 μV, SD=1.91, seizure: M=-0.43 μV, SD=2.30; t(23)=-0.53, p=0.604, d=-0.107) for the early time frame.

In contrast, for the later time frame (455-595 ms after R-wave), no significant differences between the conditions were seen (F8 baseline M=-0.58 μV, SD=1.84, seizure: M=-0.21 μV, SD=1.54; Fz baseline: M=-0.33 μV, SD=1.54, seizure: M=0.46 μV, SD=2.46; F7 baseline: M=-0.11 μV, SD=1.47, seizure: M=0.58 μV, SD=2.72, all p’s>0.05).

### Control analysis of ECG data

To check whether differences in HEPs between conditions were related to differences in the ECG signal recordings, mean amplitudes in ECG channels were extracted and entered in the ANOVA with factors time frames and conditions. As expected, the ANOVA showed a main effect of time frame (F(1,23) = 5.66, p=0.026, partial η²=0.198), but no main effect of condition or interaction with condition occurred.

### Correlations between HRV measures and HEPs

**Figure 3:**
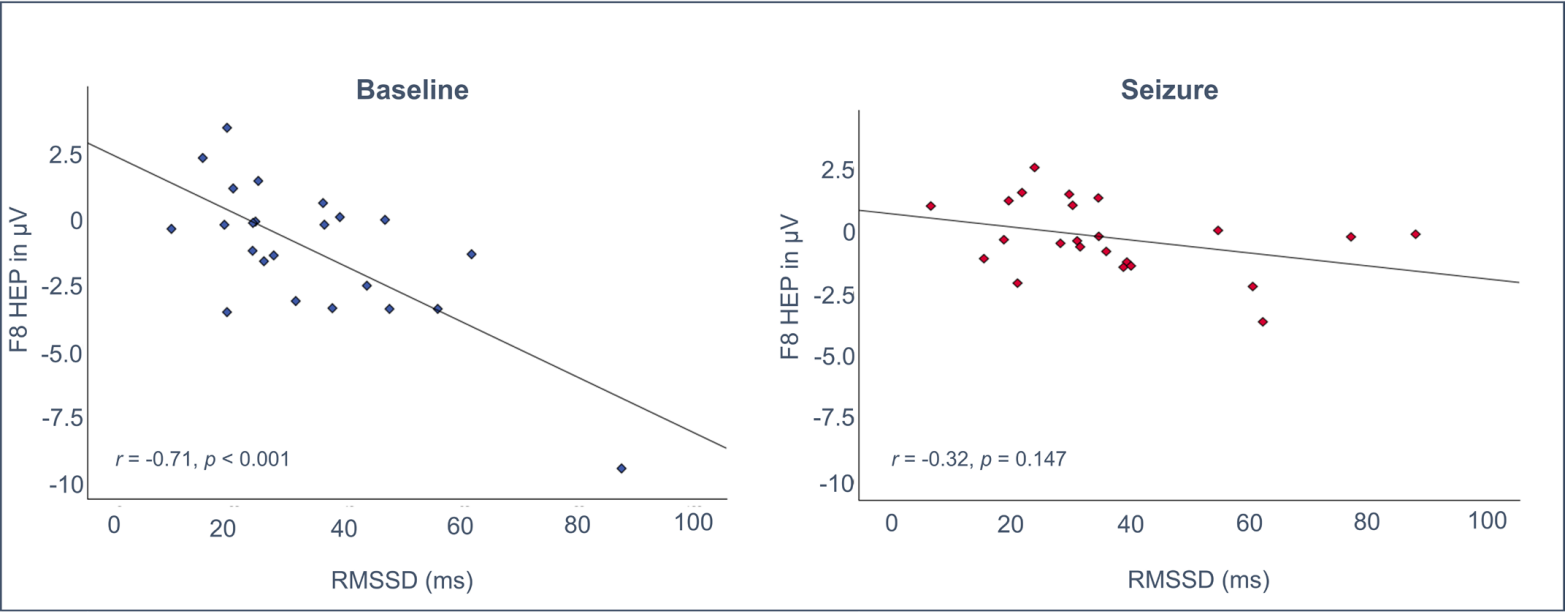
Exemplary illustration of the correlation between heart rate variability (RMSSD, root mean square of the successive differences; a measure of short-term variability in heart rate reflecting parasympathetic activity) and heartbeat-evoked potentials (F8) at baseline (left) and during seizures (right). During seizures, correlations were weakened and lost statistical significance. HEP, heartbeat-evoked potential.

Partial correlations (controlling for the mean amplitude in ECG channels) during the baseline recording reached significance for HEP over F8 and Fz, and HRV data across parameters. Since the polarity of the HEPs over F8 is negative, more negative HEP amplitude values mean more pronounced potentials, and correlations have to be interpreted with this in mind. During seizures, correlations were weakened and mainly lost statistical significance (table 2, figure 3).

**Table 2:** Correlations (r (p)) of HRV variables with heartbeat-evoked potentials (HEP) at baseline and during the seizure

	HR	PNS index	SNS index	SDNN	RMSSD
Baseline HEP					
F7	0.377 (0.084)	-0.26 (0.248)	0.38 (0.084)	-0.34 (0.124)	-0.26 (0.243)
F8	**0.46** (**0.023**)	-0.61 (0.003)*	**0.54** (**0.009**)	**-0.63** (**0.002**)*	-0.71 (<0.001)*
Fz	**0.45** (**0.036**)	**-0.46** (**0.033**)	**0.43 (0.048)**	-0.64 (0.001)*	**-0.56** (**0.007**)
Seizure HEP					
F7	0.03 (0.903)	-0.05 (0.812)	0.05 (0.834)	0.05 (0.831)	-0.06 (0.776)
F8	0.28 (0.212)	-0.399 (0.066)	0.35 (0.110)	-0.58 (0.004)*	-0.32 (0.147)
Fz	0.03 (0.912)	-0.08 (0.723)	0.06 (0.783)	-0.18 (0.429)	-0.10 (0.642)

Significant correlations (p<0.05) are printed in bold font, correlations withstanding Bonferroni-correction are marked with an additional*.

Bonferroni correction was performed for eight variables, resulting in a significance threshold of p=0.05/8=0.00625.

HR, heart rate; HRV, heart rate variability; PNS, parasympathetic autonomic nervous system; RMSSD, root mean square of the successive differences; SDNN, SD of normal-to-normal intervals; SNS, sympathetic autonomic nervous system.

Calculation of differences between correlation coefficients showed a significant difference between conditions (baseline vs seizure) for the correlations HEP-F8-RMSSD (*z*=-1.71, p=0.043) and HEP-Fz-SDNN (*z*=-1.79, p=0.037) and a tendency for HEP-Fz-RMSSD (*z*=-1.63, p=0.052, all other p’s>0.05).

We tested whether individual changes from baseline to seizure in HEP amplitude and HRV were related. There were no statistically significant correlations (eg, Δ HEP F8 with Δ RMSSD: r=-0.1, p=0.329; Δ HEP F8 with Δ PNS Index: r=-0.118, p=0.591; for Fz and F8 all p’s>0.05).

### Correlations between HEPs and cortical thickness

**Figure 4:**
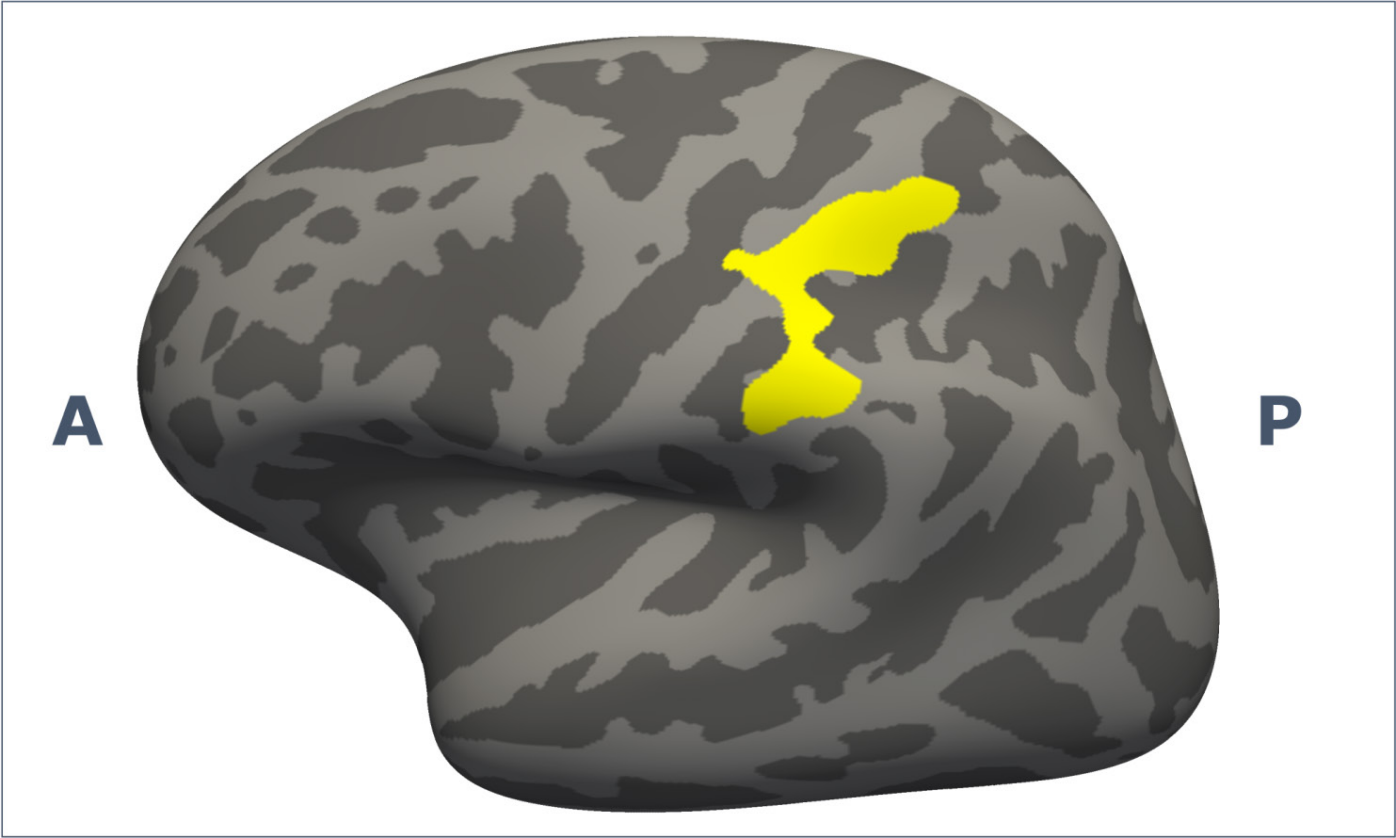
Correlation of cortical thickness with early central (Fz) heartbeat-evoked potential amplitude, corrected for age, sex, scanner type and total intracranial volume. A, anterior; P, posterior.

To investigate whether HEP amplitudes were related to cortical thickness, whole-brain/whole-hemisphere vertex-vise correlation analyses with HEP amplitudes were performed. Early central (Fz) HEPs during seizures were positively correlated (p=0.003) with cortical thickness of the left supramarginal (somatosensory association cortex; figure 4). Other correlations between early or late HEPs in F7 and F8 with cortical thickness were not observed.

## Discussion

In this retrospective study, we sought to investigate the interplay between autonomic arousal (measured using HR and HRV) and interoception (assessed through HEPs) during dissociative seizures. We identified significantly higher autonomic arousal during seizures compared with baseline. (Right) frontal HEP amplitudes were significantly smaller during seizures compared with baseline for the early time frame (250-455 ms after R-wave). This suggests alterations in the cortical representation of interoceptive information during dissociative seizures. Importantly, those changes in HEPs were not related to differences in the ECG signal, demonstrating that alterations in HEP are not a mere reflection of changes in signal strength. HEP amplitude was correlated with a range of HRV measures at baseline (higher HEP amplitude with higher autonomic arousal), but this association was largely lost during seizures. Potentially, this highlights a disintegration of autonomic arousal measures and interoceptive processing during dissociative seizures, reflecting the reduction in body awareness as a core dissociative ictal phenomenon. However, we did not see a correlation between individual changes in HEPs and HRV, so the loss of group-level correlation might have other reasons. HEP amplitudes were correlated with cortical thickness of the somatosensory association cortex, suggesting a neuroanatomical volumetric correlation of electrophysiological interoceptive signals.

Overall, our study confirms previous ictal HR and HRV findings of heightened autonomic arousal, as similar results were reported by others; this overall tendency to heightened autonomic arousal at the onset of dissociative seizures is also reflected in a recent meta-analysis.^11^ Regarding HEPs, the only study to date in dissociative seizures compared immediately preictal to interictal states^23^ while we compared preictal or interictal baseline to seizure states. In their study, Elkommos *et al* found a reduction in HEP amplitude during preictal compared with interictal states in right frontal and central electrodes. Even though we looked at data from the beginning of seizures and not just before it, due to the inherent difficulty of clearly delineating a precise time point of onset in dissociative events, both findings can be seen as in accordance.

As mentioned, one possible interpretation of the ictal loss of group-level correlation between HRV and HEP is a disintegration of arousal and interoceptive processing during dissociative seizures. In other clinical populations (derealisation/depersonalisation disorder and borderline personality disorder), a similar relationship between autonomic arousal and HEPs has been reported.^20 24^ However, there is significant heterogeneity in methods and findings, making firm conclusions on the relationship between arousal and HEPs difficult. In principle, the validity of assessing interoceptive processes using HEPs has not been fully determined, although meta-analyses show a moderate to large association between arousal and HEPs and a moderate association between HEP amplitude and interoception.^30^

We found correlations between HEP amplitude and cortical thickness in the left supramarginal gyrus. To our knowledge, this study is the first to investigate cortical thickness correlates of interoceptive processes in dissociative seizures. Interestingly, cortical thickness of the left supramarginal gyrus has also been reported to correlate with illness duration in patients with dissociative seizures and cortical volume of the right supramarginal gyrus was related to functional symptom severity in patients with functional movement disorders.^27 33^ Resting-state functional connectivity of the supramarginal gyrus was related to anxiety symptom severity in patients with dissociative seizures and traumatic brain injury and was among the most discriminative features when comparing patients with mixed FND to healthy controls.^34 35^

When integrating our findings into the larger picture of dissociative seizure pathophysiology, we hypothesise that the heightened autonomic arousal leads to a reduction in interoceptive processing, which in turn reduces body awareness. Body awareness is mediated by posterior-mesial regions including the posterior cingulate cortex and the precuneus,^36^ which have been found to be altered or related to illness characteristics in patients with dissociative seizures and other forms of dissociative symptoms.^27 37^ This interpretation would be in line with illness models centring around the role of interoceptive and affective mind-brain-body processes.^38^ However, our correlation results do not allow any causal conclusions to be drawn about the underlying pathomechanisms in the occurrence of dissociative seizures. Furthermore, the role of interoceptive processes may vary among patients with dissociative seizures and the broader category of FNDs, just as different pathophysiological models emphasise different aspects, usually but not necessarily including notions of emotional processes as ‘breaking points’ in pathogenesis.^39 40^

As a retrospective pilot project, this study has relevant limitations. First, we rely on a convenience sample with data originally collected for clinical purposes that did not adhere to research-level standards of completeness/consistency (eg, clinical information on seizure frequency was not available for some patients) and technical fidelity. Recorded seizures include both habitual and provoked events and the clinical documentation was incomplete in some cases. In the absence of objective criteria for seizure onset, varying indicators had to be used to mark the onset of recorded events. This and other factors led to partially inconsistent segmentation of the vEEG data (especially for the baseline condition). This is particularly relevant for the interpretation of some markers of arousal; for example, the SDNN is prone to bias when comparing segments of different lengths. Finally, even though we found no statistically significant differences in the mean ECG amplitude in the time frames of interest and controlled for ECG amplitude in our partial correlations, the morphology of the HEPs suggests that the effect might still be confounded by arousal-related changes to the ECG signal (typically increase in R-peak and decrease in T-wave). Not all included MRIs were of research-grade quality, even though the processing technique used here has been validated to work on MRI data of any modality, contrast and resolution.

In conclusion, our pilot investigation of archived EEG and ECG recordings of dissociative seizures shows an increase in sympathetic arousal, a decrease in parasympathetic activity and a decrease in HEPs over right prefrontal electrodes with baseline correlations between these measures largely disappearing ictally. These findings support an objectifiable link between arousal and diminished body awareness hypothesised to underly dissociative seizures. Correlations with HEP amplitudes were found in the left supramarginal gyrus.

## Data Availability

Data are available on reasonable request. Primary vEEG and MRI data cannot be made available, as it contains identifiable personal patient information. Other data are available in anonymised form on request from qualified researchers.
