# Supplementary material for: Heartbeat evoked potentials and autonomic arousal during dissociative seizures: insights from electrophysiology and neuroimaging
Source: BMJ Neurol Open. 2024 Jun 5;6(1):e000665. doi: 10.1136/bmjno-2024-000665 (PMC11163632; doi:10.1136/bmjno-2024-000665)
Supplement: Supplementary data [file bmjno-2024-000665supp001.pdf]

Supplementary Material

Heartbeat evoked potentials and autonomic arousal during dissociative seizures – insights from electrophysiology and neuroimaging

Vera Flasbeck[1], Johannes Jungilligens*[2], Isabell Lemke[2], Jule Beckers[2], Hilal Öztürk[2,3], Jörg Wellmer[4], Corinna Seliger[2], Georg Juckel[1], Stoyan Popkirov[5]

1 Division of Clinical and Experimental Neurophysiology, Department of Psychiatry, Psychotherapy and Preventive Medicine, LWL University Hospital Bochum, Ruhr University Bochum, Bochum, Germany

2 Department of Neurology, University Hospital Knappschaftskrankenhaus, Ruhr University Bochum, Bochum, Germany

3 Faculty of Psychology, Ruhr University Bochum, Bochum, Germany

4 Ruhr Epileptology, Department of Neurology, University Hospital Knappschaftskrankenhaus, Ruhr University Bochum, Bochum, Germany

5 Department of Neurology, University Hospital Essen, Essen, Germany

Supplementary Table 1. Demographic and clinical characteristics.

**Patient ***	**Illness duration (years)**	**Seizure frequency**	**Seizure semiology ****	**Medication**	**Included in MRI analysis**
29 F	17	yearly	initially **tingling sensation** and feeling of weakness in both legs, then symmetrical high-frequency low-amplitude trembling and shaking of both legs	none	
34 F	1	daily	feeling of tension in the legs, **headache, irregular breathing**, shoulders pulled up, whole body tensing up, head reclined, **tearful crying**, pressed voice, feeling of "deep sadness", sustained responsiveness	levetiracetam	
19 F	1	monthly	irregular involuntary jerks and thrashing movements of the left extremities, some jerks with right arm, incomplete opisthotonos	none	
32 TM	19	daily	**palpitations, tingling sensations** in left hand, eyelid flutter, **nausea**, trembling of right leg, head reclination, **tingling** of head and right body	ethylphenidat, hydroxychloroquine	Yes
19 F	9	weekly	**heat and tingling sensations of the body**, then slight trembling and then back-and-forth movement of arms and legs, incomplete opisthotonos, sustained responsivity throughout	none	Yes
20 M	1	unclear	varying convulsions of the entire body with stuttering and various **tingling** and painful sensations, no change in alertness or responsiveness	lamotrigine	Yes
59 F	4	unclear	nodding and trembling of the right hand and then left leg, intensification and generalisation of convulsions to affect whole body, then loss of verbal responsivity and incomplete memory, cued recall of questions; later reports feeling "far away" during seizure	levetiracetam, promethazine, pregabalin, venlafaxine, metformin, sitagliptin, bisoprolol, ramipril, hydrochlorothiazide, simvastatin, pantoprazole	Yes
17 F	5	unclear	irregular tremor of the right hand, feeling of warmth, **accelerated breathing, tingling sensation in the feet**	none	
39 F	1	daily	**tongue numb**, inability to speak, responsiveness retained, eyes closed, slight jerks of right arm/shoulder, becomes unresponsive to verbal and painful stimuli, unwell and exhausted afterwards	acetylsalicylic acid	Yes
42 M	26	daily	back-and-forth rocking/thrashing of head, then also involving the arms, wheezing sounds, trembling of jaw, unresponsive, fluctuating course	l-thyroxine, metamizole, domperidone, pantoprazole	Yes
59 M	2	unclear	minor motor **restlessness** with intermittent jerks of the head and both legs, alert throughout	pantoprazole, torasemide, allopurinol, ezetimibe, simvastatin, acetylsalicylic acid, amlodipine, candesartan	Yes
60 M***	<1	monthly	painful spasms of the right hand and slowing of speech, absence-like intermittent loss of awareness with eyes rolled upwards, reports no memory of events	atorvastatin, acetylsalicylic acid, citalopram, ramipril, metoprolol, tamsulosin, tizanidine, baclofen, cannabis	Yes
52 F	<1	unclear	high-frequency low-amplitude tremor of the right hand and left leg, later also involving left hand, spasms in the shoulders and the jaw, stuttering speech	pregabalin, omeprazole, amlodipine, indapamide, ramipril, l-thyroxine, lithium, celecoxib, venlafaxine, zolpidem, buprenorphine, metoclopramide	Yes
42 F	<1	weekly	**feeling of unease, chest tightness, anxiety**, eyelid flutter, no reaction to speech, eyes squeezed shut upon passive opening attempt, afterwards feeling of heaviness in the left arm and a tremor on the left side	bisoprolol, l-thyroxine	Yes
19 F	7	weekly	closes eyes, clenches hands into fists, bends at elbows, does not respond (10 minutes), opisthotonos and generalized tremulous movements, loss of responsiveness	levetiracetam, oral contraceptive (unspecified)	

66 F	1.5	daily	after hyperventilation exercise prolonged loss of responsiveness for several minutes	none	Yes
36 F	4	monthly	feeling of paralysis, slowing of movements and speech, **warming sensation**, impaired responsiveness for several minutes	lamotrigine, levetiracetam, zopiclone	
21 F	2	unclear	impairment of alertness, reduced responses, whispers, reports blurred vision, eyelid flutter	levetiracetam	
40 F	13	daily	brief episodes of complex and bizarre movements of the arms and legs, head jerks back-and-forth, intermittent vocalization, eyes open and close	pregabalin, quetiapine, risperidone, venlafaxine	Yes
27 F	10	unclear	sudden lifting of the left arm, looks around the room with wide open eyes, no adequate response to address, left hand held with fingers splayed, slight turning of the head towards person addressing her, the upper body is jerked backwards several times and the whole body is tensed (opisthotonos-like movement), hands held in claw position	none	
40 M****	2	unclear	**restlessness/nervousness**, occipital piercing headache, mixed tremulous and myoclonic movements of the left arm	promethazine, pregabalin	
55 F	19	weekly	**tingling sensation of the lower lip and right hand**, turns head to the right and it falls limply to the right, spreads her left arm supporting on the back of the chair, eyes closed, unresponsive to speech, slowed response to pain stimuli, **tingling sensation of the whole body**, slight toning of the arms and legs with recurrent slight jerks of the legs upward and adduction, fingers in claw posture, reports **'funny head feeling', nausea** and **dizziness** ictally, **breathes faster** and with difficulty, somewhat fatigued afterwards	lamotrigine, citalopram, etoricoxib	Yes
21 F	4	monthly	**feeling of warmth**, subjective paralysis, loss of feeling in lower body, **tingling sensation in the head**	lamotrigine, l-thyroxine, oxybutynin	Yes
19 M	6	unclear	high-frequency back-and-forth movement of the left arm, then feeling of **tightness and pain in the chest**, responsiveness unimpaired	none	Yes
18 F	1	unclear	loss of responsiveness, eyelid flutter, irregular opisthotonos, grimacing, upper body rocking motions, dystonic posturing oft both arms/hands as well as jaw, sobbing, fluctuating course with brief intermittent responsiveness	none	Yes

Notes:
*Patients characterized by age in years and gender; F, female; M, male; TM, transgender male.
** Semiological features potentially related to autonomic arousal highlighted in bold.
*** Patient was excluded from HRV-analysis as he had a pacemaker.
**** Patient was excluded from heartbeat-evoked potentials and MRI analyses as he had a large arachnoid cyst (left fronto-temporal).

Supplementary Methods – Analysis of cortical thickness data

Supplementary Table 2. Scanner and sequence details

No of subjects	Manufacturer	Model	Field strength	Slice thickness	Echo time	Repetition time
6	Siemens	Prisma	3	1	0.00229	2.2
4	Siemens	Prisma	3	0.85	0.00239	1.8
1	Philips	Achieva	1.5	1.6	0.004838	2.5
1	Siemens	Aera	1.5	1	0.00288	2.2
1	Siemens	Altea	1.5	1	0.00244	0.9
1	Siemens	Altea	1.5	1	0.00279	2.2
1	Siemens	Vida	3	0.9	0.00232	1.69
1	GE	Signa HDxt	3	2.5	0.099008	5.2

DICOM images were converted to nifti format using dcm2niix. The FreeSurfer 7.4.1 recon-all-*clinical* pipeline was used. This new tool enables performing the FreeSurfer cortical reconstruction on clinical-grade MRI scans irrespective of imaging quality. It entails several extra procedures not part of the usual recon-all pipeline, including obtaining a volumetric segmentation and linear registration to Talairach space, synthetization of a a higher resolution 1mm T1 image, and predicting the distance maps and reconstructing topologically accurate cortical surfaces to fit surfaces. Details can be found online: https://surfer.nmr.mgh.harvard.edu/fswiki/recon-all-clinical

The other processing steps are equivalent to the standard recon-all pipeline and include automatic cortical reconstruction, surface inflation, registration to FreeSurfer's default average spherical atlas and parcellation. To obtain cortical thickness measurements, the closest distance from the white/grey boundary to the grey/cerebrospinal fluid boundary was calculated individually at each point across the cortical mantle. Following this, the parcellation of the cortical surface in anatomically distinct regions was based on the Desikan-Killiany parcellation scheme which is FreeSurfer's default atlas.

Outputs were visually inspected for quality and accuracy. Prior to statistical analyses, a 10-mm full-width at half-maximum Gaussian Kernel was applied to cortical thickness maps.

Supplementary Methods – Additional Results of Heartbeat-Evoked Potentials

The ANOVA for central electrodes revealed again a main effect of timeframe ($F(1,23) = 4.77$, $p = 0.039$, partial $\eta^2 = 0.172$) and an interaction of timeframe with condition and electrode ($F(1.76, 40.50) = 5.32$, $p = 0.011$, partial $\eta^2 = 0.188$). Post-hoc tests for the interaction showed a significant difference between conditions only for the Cz electrode in the early timeframe (250-455 ms: Cz seizure: $M = 0.19$ µV, $SD = 1.74$, baseline: $M = -0.76$ µV, $SD = 2.21$; $t(23) = 2.22$, $p = 0.036$, $d = 0.454$; C3 seizure: $M = 0.09$ µV, $SD = 1.49$, baseline: $M = -0.52$ µV, $SD = 2.01$; $t(23) = 1.90$ $p = 0.070$, $d = 0.388$; C4 seizure: $M = 0.11$ µV, $SD = 1.64$, baseline: $M = -0.69$ µV, $SD = 1.97$; $t(23) = 2.00$, $p = 0.057$, $d = 0.408$; for 455-595 ms all p's > 0.05). When using electrodes F3 and F4 instead of F7 and F8, the ANOVA showed again the main effect of timeframe ($F(1,23) = 11.11$, $p = 0.003$, partial $\eta^2 = 0.326$) and the interaction of timeframe with condition and electrode ($F(1.40, 32.13) = 4.86$, $p = 0.024$, partial $\eta^2 = 0.174$) indicating a difference between conditions over F4 (and Fz) for the early timeframe (250-455 ms: F4 seizure: $M = 0.09$ µV, $SD = 2.20$, baseline: $M = -1.44$ µV, $SD = 2.59$; $t(23) = 2.15$, $p = 0.042$, $d = 0.439$; Fz baseline: seizure: $M = 0.20$ µV, $SD = 2.16$, baseline: $M = -1.15$ µV, $SD = 2.36$; $t(23) = 2.285$, $p = 0.032$, $d = 0.466$; for 455-595 ms all p's > 0.05). The ANOVA for frontopolar electrodes (Fp1, Fp2 and Fpz) showed the main effect of timeframe ($F(1,23) = 13.35$, $p = 0.001$, partial $\eta^2 = 0.367$), but no interaction. For HEP waveforms see Figure S1.

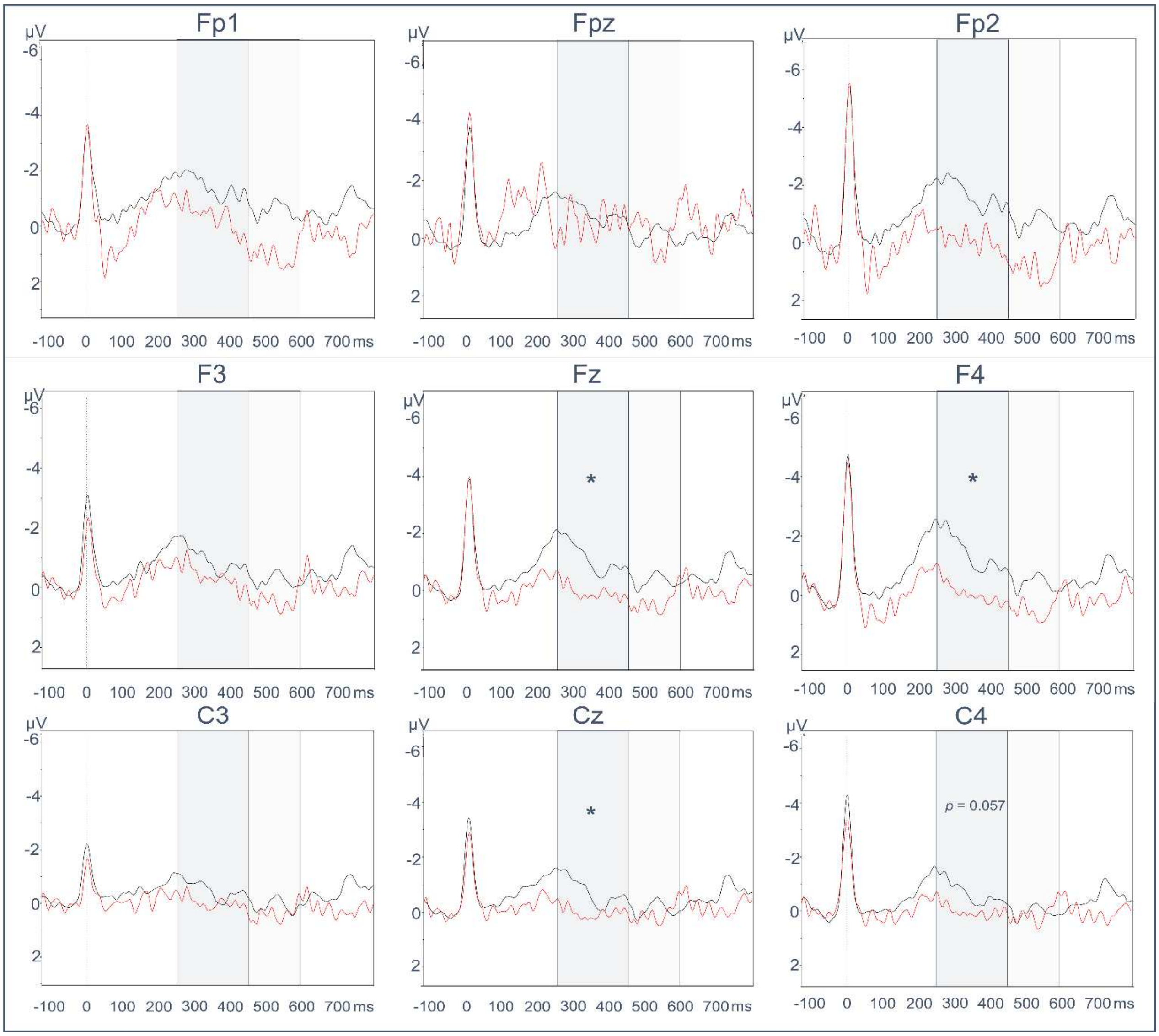

Figure S1. *Comparisons of heartbeat-evoked potential (HEP) waveforms during baseline (black) and seizure (red) for Fp1, Fpz and Fp2 (upper row) electrodes, F3,Fz and F4 electrodes (middle row) and C3, Cz and C4 (lower row) electrodes. * $p < 0.05$*
